# Chinese Seed Trait Database: a curated resource for diaspore traits in the Chinese flora

**DOI:** 10.1111/nph.70296

**Published:** 2025-06-12

**Authors:** Hao‐Yu Wang, Xue‐Lin Chen, Si‐Chong Chen

**Affiliations:** ^1^ State Key Laboratory of Plant Diversity and Specialty Crops Wuhan Botanical Garden, Chinese Academy of Sciences Wuhan 430074 China; ^2^ University of Chinese Academy of Sciences Beijing 101408 China; ^3^ College of Life Science Nanchang University Nanchang 330031 China; ^4^ Royal Botanic Gardens Kew Surrey TW9 3AE UK

**Keywords:** China, geographical bias, open science, regeneration, seed dispersal, seed germination, seed mass, seed size

## Disclaimer

The New Phytologist Foundation remains neutral with regard to jurisdictional claims in maps and in any institutional affiliations.

## Introduction

Regeneration marks the beginning of a plant's life cycle, with profound effects on population recruitment, species adaptation, and community resilience to environmental changes (Grubb, 1977). Diaspores, including seeds, fruits, and their appendages (hereafter ‘seeds’), are crucial agents of plant regeneration, possessing a wide range of functional traits related to dispersal and colonisation (Saatkamp *et al*., 2019). Trait‐based approaches have been widely used to investigate responses of plants to both biotic and abiotic conditions from individual to ecosystem scales (Díaz *et al*., 2016; He *et al*., 2019). To date, traits of vegetative organs are overrepresented in existing global and regional databases, largely due to the essential roles of leaves in ecological functions and ecosystem services as well as the relative ease of accessing and measuring these traits (Supporting Information Table S1). However, our understanding of plant traits remains incomplete, particularly for those traits that are difficult to measure, such as regenerative traits (known as the ‘Raunkiæran shortfall’; Hortal *et al*., 2015). Several databases have made efforts to address this gap (Table S1). For instance, the TRY database has compiled over 50 distinct seed traits with decent species representation (Kattge *et al*., 2020), and the GIFT database has also aggregated substantial seed trait data (Weigelt *et al*., 2020). Some regional databases, such as Rasgos‐CL (Alfaro *et al*., 2023) and LEDA (Kleyer *et al*., 2008), with over 20% seed trait records, have contributed to this field. Nevertheless, seed trait data remain relatively sparse both in terms of trait diversity and record coverage (Table S1). Advancing key research questions in plant science necessitates comprehensive seed trait data, along with associated geographic information (Saatkamp *et al*., 2019). These demands have prompted the recent initiation of several databases focusing on seed germination (Fernández‐Pascual *et al*., 2023), seed dormancy (Rosbakh *et al*., 2020), and Brazilian rock vegetation seed traits (Ordóñez‐Parra *et al*., 2023). Despite these advances, seed‐related data lag far behind those centred on vegetative traits in existing databases (Larson & Funk, 2016; Kattge *et al*., 2020).

Current research efforts remain disproportionately focused on specific plant communities or habitats within certain regions, leading to heavily regional biases in the current global coverage of seed traits (Silveira *et al*., 2023). China, which has a wide range of biomes, is renowned for its remarkable floral diversity of over 35 000 vascular plant taxa (Lu & He, 2017). For example, the Qinghai‐Tibet Plateau, known as the ‘Roof of the World’, harbours at least 12 000 plant species, of which almost 20% are endemic to the region (Wen *et al*., 2014). These abundant resources have enabled a wealth of research by Chinese scholars, establishing China as a growing contributor to the international plant science community. According to the Chinese Science Citation Database, > 1000 authorised journals in science and technology are published in Chinese (http://sdb.csdl.ac.cn; Jin & Wang, 1999). However, due to the language barrier and access limitation, this wealth of information remains a rich yet underutilised resource for plant science studies in the international research community (Amano *et al*., 2016). To date, a number of regional databases have emerged, for example, AusTraits summarises data on plant traits in Australia (Falster *et al*., 2021), and FunAndes compiles trait data on major plant taxa in the tropical Andes (Báez *et al*., 2022). Integrating regional databases with global ones can enhance the completeness of trait data and mitigate the geographical biases in global data coverage (Maitner *et al*., 2023). Unfortunately, limited progress has been made in systematically compiling regional data on seeds. Here, we present a curated repository that compiles an extensive array of seed traits for plant species in China – the Chinese Seed Trait Database (hereafter ‘CSTD’; https://macroeologygroup.shinyapps.io/CSTD). Our goal is to address gaps in current plant trait data and to enhance the accessibility of seed trait data to the international research community.

## Current state of the Chinese Seed Trait Database

The CSTD has so far been assembled from 694 sources in the Chinese language, including 681 journal papers, 10 books, and three online datasets. In total, the CSTD comprises 110 451 records across 118 distinct seed traits for 3897 species in 1416 genera and 214 families, covering a broad range of climates and biomes with most records geo‐referenced. We conducted an extensive literature search in the China National Knowledge Infrastructure and the Taiwan Airiti Library, using the keywords ‘seed’ and ‘trait’ in Chinese. The search included published literature from 1980 to 2024, ultimately resulting in a total of 8443 papers, of which 681 were identified as containing seed trait data. Meanwhile, we digitised data from 10 books, which were published by Chinese scholars based on their specific work. We also acquired relevant data from three online datasets in Chinese deposited in the Plant Science Data Centre (https://www.plantplus.cn/), Science Data Bank (http://www.scidb.cn), and Global Change Research Data Publishing & Repository (https://geodoi.ac.cn/WebEn/Default.aspx).

**Fig. 1:**
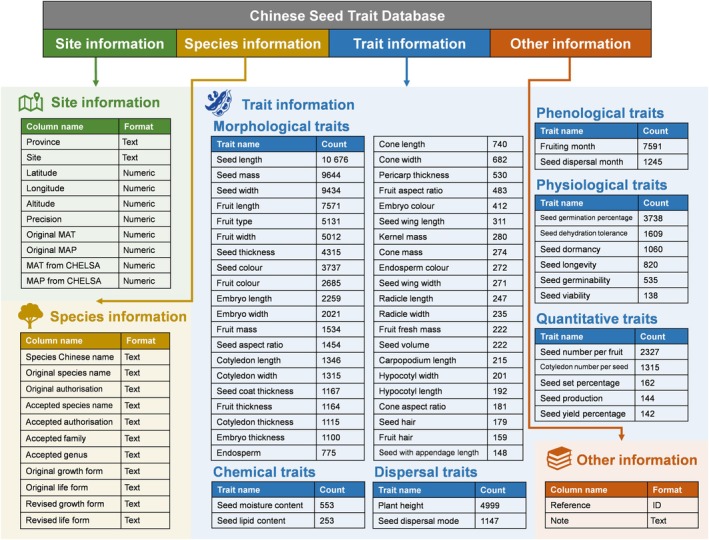
Structure of the Chinese Seed Trait Database. The database consists of four sections. Site information (in green) is in the first 10 columns of the table, followed by 11 columns of species information (in yellow). Trait information (in blue) is classified into six categories (morphological, quantitative, phenological, physiological, chemical, and dispersal). Other information (in orange) contains reference and note in two columns of the table. The main traits with more than 140 records are listed here, whereas a full list of 118 traits is provided in Supporting Information Table S2. CHELSA, climatologies at high resolution for the earth's land surface areas; MAP, mean annual precipitation; MAT, mean annual temperature.

The database is structured as a single table, divided into four sections according to column headings (Fig. 1): (1) site information, such as sampling location, coordinates, and macroclimate; (2) species information, such as taxonomy, growth form, and life form; (3) trait information, such as trait name, trait value, value type, and unit and (4) other information, such as reference and note.

### Site information

**Fig. 2:**
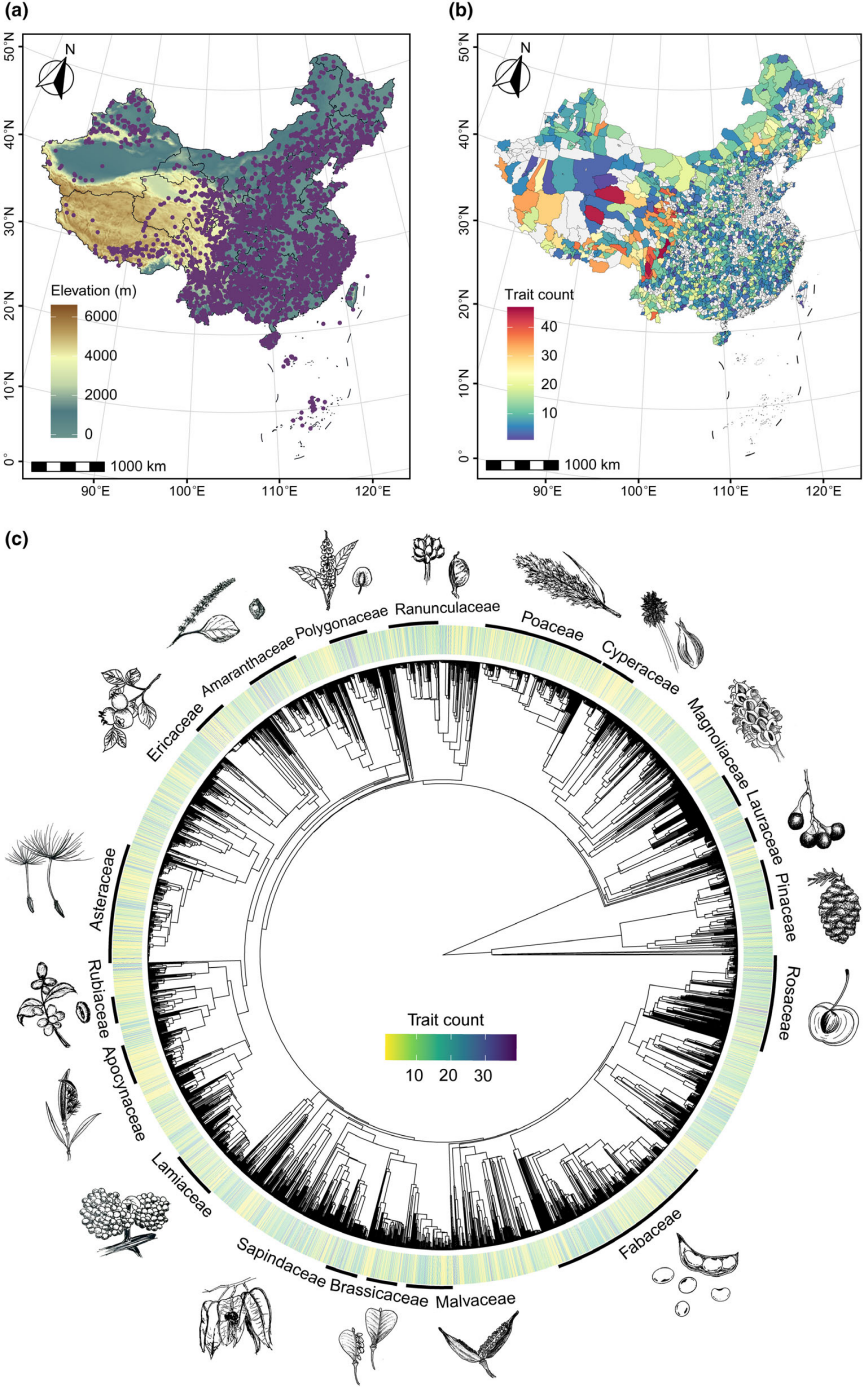
Trait coverage across geography and phylogeny in the Chinese Seed Trait Database. (a) Locations of geo‐referenced records. (b) County‐level distribution of trait counts. (c) Trait count per species projected on a phylogeny sourced in the ‘V.PhyloMaker’ R package (Jin & Qian, 2022), labelled with families that have > 50 species in the database.

We have incorporated site information whenever possible, to allow users to access both geographic and trait information on species. We retrieved detailed coordinates (WGS84) and corresponding climate data (mean annual temperature and precipitation) from the original sources whenever possible. Otherwise, for records with toponym below the county level lacking coordinates, we obtained coordinates and elevations through Google Earth or supplemented elevations using the R package elevatr (Hollister, 2023). Additionally, we extracted key climate variables from the high‐precision CHELSA dataset for user convenience (Karger *et al*., 2017), while the provided coordinates enable users to retrieve other environmental data. Overall, 79% of the records in the CSTD include toponym information, among which 66.3% have precise coordinates at county level or finer spatial resolutions (i.e. including specific information on latitude, longitude, and elevation). These geo‐referenced records span a broad range across all provinces of China, with an elevational gradient from 0 to 6000 m and a latitudinal gradient from 7.4°N to 52.9°N (Fig. 2a). Notably, in addition to the extensive mainland data, 17% of the records are from islands (Fig. 2a). Counties in the Qinghai‐Tibet Plateau and the Hengduan Mountains exhibit the greatest diversity of trait types, despite most data being for eastern China (Fig. 2b). The extensive geographic coverage in the CSTD enables access to environmental and biotic data from additional sources, and thereby enhances efforts to investigate large‐scale patterns and the underlying mechanisms.

### Species information

In most cases, source papers provided both Chinese and Latin names, which we standardised to accepted names using the World Checklist of Vascular Plants via the R package taxize (Chamberlain *et al*., 2020). For sources reporting only Chinese names, we manually converted them to Latin binomials according to the Flora of China before proceeding with standardisation. We maintained the Chinese names for species, to make the CSTD a valuable resource for both international and local researchers. Plant growth forms and life forms were categorised and matched primarily based on the dataset compiled by Zheng *et al*. (2024). For instance, growth forms were classified based on stem lignification as herbaceous or woody, with woody plants further divided into trees and shrubs according to trunk presence and height. In total, trait coverage is relatively even across the phylogeny, with a substantial portion of species (38.7%) documented with more than 10 distinct traits, presenting comprehensive profiles (Fig. 2c). Fabaceae, Asteraceae, Poaceae, and Rosaceae have the highest species representation (Fig. 2c). However, certain species‐rich families, such as Orchidaceae and Caryophyllaceae, remain underrepresented, highlighting the need for further research on their seed traits (Fig. 2c). Species in the CSTD represent nearly all growth and life forms, with medium trees and deciduous species predominating among woody plants, and forbs and perennials predominating among herbaceous plants (Fig. S1). Therefore, the broad taxonomic coverage and diversity of plant forms provide a strong foundation for large‐scale phylogenetic comparative analyses of seed traits, which allow us to quantify the role of seed trait syndromes in plant diversification.

### Trait information

We compiled 102 continuous and 16 categorical traits, adhering to the standard definition of ‘trait’ as a stable and measurable property of an organism that reflects its response to environmental variations (Violle *et al*., 2007). Although morphological traits dominate the database (76% of all records), the CSTD also includes a range of quantitative, phenological, physiological, chemical, and dispersal traits, which together account for *c*. 24% of all records, including well‐documented traits, such as fruiting month, seed germination percentage, seed number per fruit, and seed dehydration tolerance (Fig. 1; Table S2). These traits, which are underrepresented in global databases such as TRY and GIFT, span all major axes of the seed trait spectrum, thereby enhancing efforts to quantify the seed functional trait spectrum (Saatkamp *et al*., 2019). Continuous traits comprise the majority of the CSTD, accounting for 76% of the records. We have retained the measurement units used in the data sources, so users should be aware of potential unit inconsistencies and the necessity for unit standardisation. Notably, 10 traits – seed length, seed mass, seed width, seed thickness, fruit length, fruit width, fruiting month, plant height, seed number per fruit, and seed germination percentage – account for more than half of the CSTD records. These traits span 4–10 orders of magnitude; for example, seed mass exhibits the greatest variation, ranging from 0.00006 mg to 157 000 mg (Fig. S2). However, certain traits, such as seed nutrient contents (fewer than 25 records), remain severely underrepresented despite their importance, highlighting the urgent need for further research. The broad ranges of trait values and types highlight the extensive diversity of strategies captured in our database, facilitating the synthesis of primary seed trait axes across ecological scales and contributing to the comprehensive mapping of the regenerative spectrum across plant species.

## Data accessibility, curation, and outlook

To enhance the visualisation of the database, we have developed the CSTD application using the shiny package (Chang *et al*., 2022), which is accessible at http://macroecologygroup.shinyapps.io/CSTD. Our database comprises a primary xlsx file, an R markdown script, and a visual application. The xlsx file, entitled *CSTD_v1.xlsx*, contains seed trait data (sheet name ‘Seed trait data’), original data sources (sheet name ‘Reference’), metadata of column names (sheet name ‘Data description’), and definitions of seed traits (sheet name ‘Trait description’). The R markdown file provides code for taxonomic standardisation and figure generation. By visiting the application, users can easily access the seed trait data as well as the original sampling location for each species. Additionally, to promote open access and facilitate data use, the CSTD files are also available in the Figshare data repository (https://doi.org/10.6084/m9.figshare.28152035). For the convenience of Chinese‐speaking users, a summary of this work in Chinese is provided in Notes S1.

We are dedicated to keeping the CSTD updated with the latest data, while continuously enhancing its accuracy and comprehensiveness. Specifically, we will continue curating the CSTD by incorporating new Chinese publications and further expanding the literature synthesis to complement relevant data from international sources. Furthermore, we are going to expand the CSTD to cover previously neglected plant lineages or seed traits through field sampling and trait measurements. We also invite the global plant science community to contribute seed trait data for the Chinese flora. Despite each record having been thoroughly checked for accuracy, some errors may still remain. We encourage users to actively contribute to the ongoing maintenance and improvement of the database by offering feedback and suggestions.

In summary, the CSTD mitigates the scarcity of seed trait data by bridging valuable knowledge hidden in literature published in the Chinese language to the whole plant science community. We envision that this work represents an important step towards a global seed trait database, hopefully fostering more robust studies in the plant sciences. Our database not only allows for in‐depth research on specific species but also provides a framework for comparative studies that can provide insights into large‐scale trends in plant regenerative strategies and their ecological consequences. The CSTD enhances the potential for cross‐taxon and cross‐geography analyses and meta‐analyses aimed at testing ecological hypotheses related to plant regeneration from seeds, yet also goes a long way towards addressing the Raunkiæran shortfall.

## Competing interests

None declared.

## Author contributions

S‐CC conceived the idea. S‐CC and H‐YW constructed the database and wrote the manuscript. H‐YW and X‐LC compiled the data. H‐YW visualised the data.

## Supporting information


**Fig. S1** Pie charts demonstrating the frequencies of plant growth form and life form.
**Fig. S2** Histograms of main traits in the Chinese Seed Trait Database.
**Table S1** Summary of seed traits in prevailing plant trait databases.
**Table S2** Seed traits in the Chinese Seed Trait Database, ordered by descending records.
**Notes S1** Chinese summary of our database.
Please note: Wiley is not responsible for the content or functionality of any Supporting Information supplied by the authors. Any queries (other than missing material) should be directed to the *New Phytologist* Central Office.

## Data Availability

The data and code used to produce this article are available via the CSTD web application at http://macroecologygroup.shinyapps.io/CSTD, which also links to the Figshare at https://doi.org/10.6084/m9.figshare.28152035.
