# Supplementary material for: Chinese Seed Trait Database: a curated resource for diaspore traits in the Chinese flora
Source: New Phytol. 2025 Jun 12;248(1):11–6. doi: 10.1111/nph.70296 (PMC12409095; doi:10.1111/nph.70296)
Supplement: Supplementary file 1 — Fig. S1 Pie charts demonstrating the frequencies of plant growth form and life form. Fig. S2 Histograms of main traits in the Chinese Seed Trait Database. Table S1 Summary of seed traits in prevailing plant trait databases. Table S2 Seed traits in the Chinese Seed Trait Database, ordered by descending records. Notes S1 Chinese summary of our database. Please note: Wiley is not responsible for the content or functionality of any Supporting Information supplied by the authors. Any queries (other than missing material) should be directed to the New Phytologist Central Office. [file NPH-248-11-s001.docx]

***New Phytologist* Supporting Information**

Article title: Chinese Seed Trait Database: A curated resource for diaspore traits in the Chinese flora

Authors: Hao-Yu Wang, Xue-Lin Chen, Si-Chong Chen

Article acceptance date: 26 May 2025

The following Supporting Information is available for this article:

**Fig. S1** Pie charts demonstrating the frequencies of plant growth form and life form

**Fig. S2** Histograms of main traits in the Chinese Seed Trait Database

**Table S1** Summary of seed traits in prevailing plant trait databases

**Table S2** Seed traits in the Chinese Seed Trait Database, ordered by descending records

**Notes S1** Chinese summary of our database


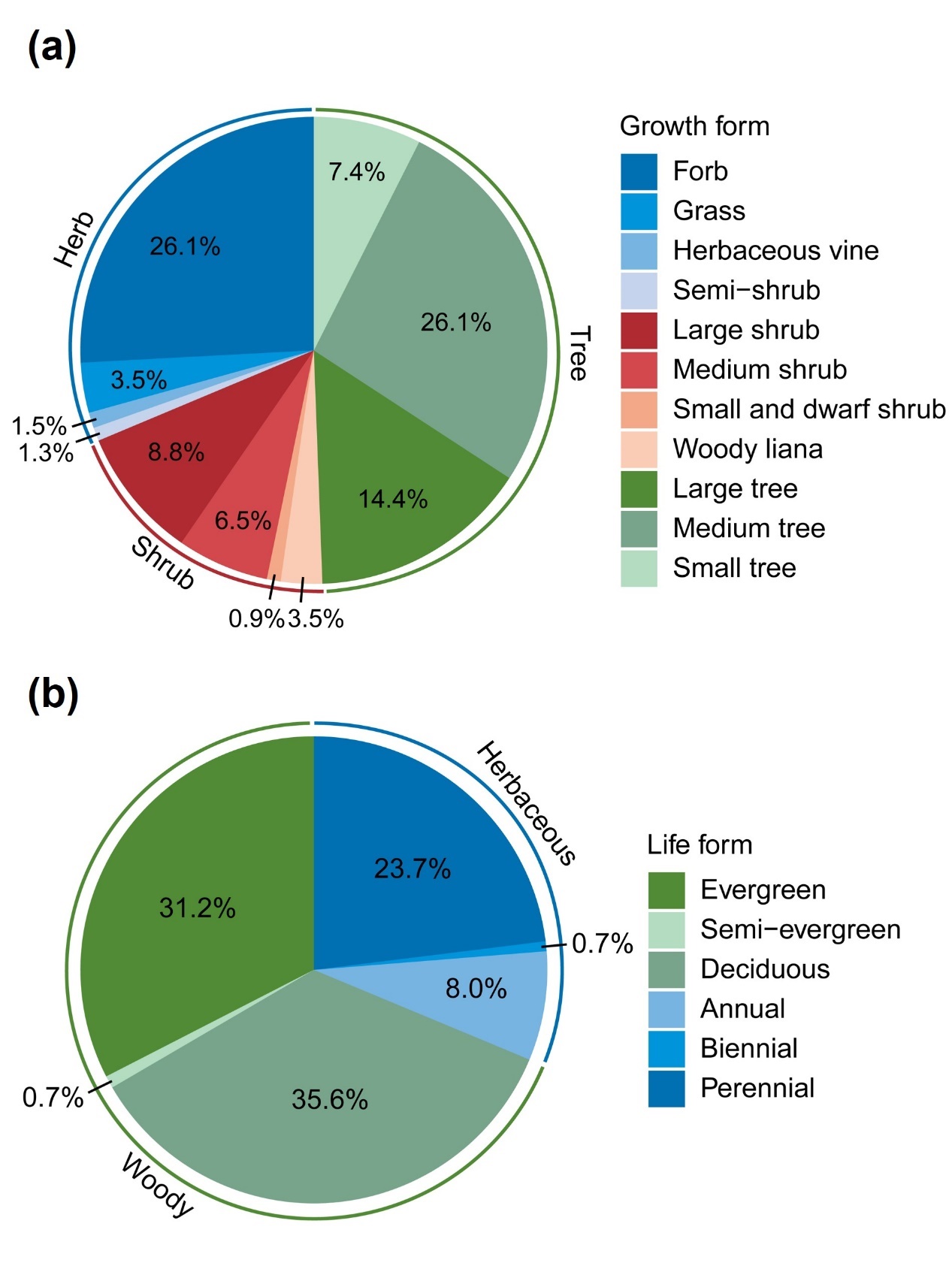


**Fig. S1** Pie charts demonstrating the frequencies of (a) plant growth form and (b) life form.


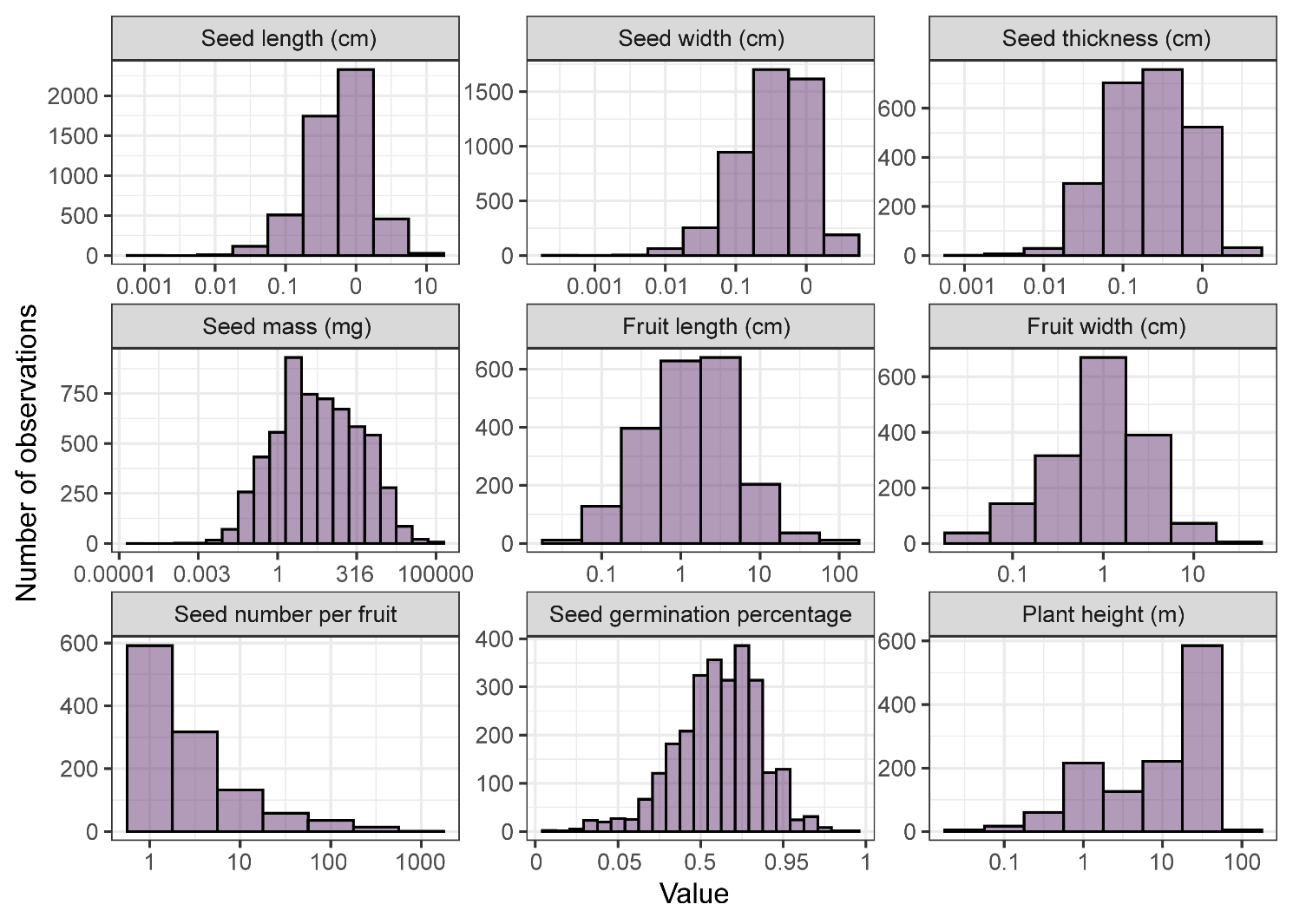


**Fig. S2** Histograms of main traits in the Chinese Seed Trait Database. Frequency distributions of observations for the well recorded continuous traits based on the mean values. (a) Seed length (cm); (b) Seed width (cm); (c) Seed thickness (cm); (d) Seed mass (mg); (e) Fruit length (cm); (f) Fruit width (cm); (g) Seed number per fruit; (h) Seed germination percentage; (i) Plant height (m). All traits except seed germination percentage are log-transformed, and seed germination percentage is logit-transformed.

**Table S1** Summary of seed traits in prevailing plant trait databases. The database column is ordered by region in descending total trait records.

| **Database** | **Region** | **Number of records** | | | **Number of traits** | | | **Number of species** | | |
| --- | --- | --- | --- | --- | --- | --- | --- | --- | --- | --- |
|  |  | **Total traits** | **Seed traits** | **Percentage** | **Total traits** | **Seed traits** | **Percentage** | **Total traits** | **Seed traits** | **Percentage** |
| **Database on plant all traits** | | | | | | | | | | |
| TRY | Global | 11,850,781 | 799,028 | 6.74% | 2,091 | 53 | 2.53% | 279,875 | > 43,227 | > 15.45% |
| GIFT | Global | 3,475,337 | 168,531 | 4.85% | 63 | 14 | 22.22% | 364,571 | > 23,874 | > 6.55% |
| BIEN | Global | 916,988 | 94,015 | 10.25% | 28 | 2 | 7.14% | 93,251 | > 9,941 | > 10.66% |
| CoRRE | Global | 306,919 | 2,926 | 0.95% | 17 | 1 | 5.88% | 4,079 | 2,629 | 64.45% |
| AusTraits | Australia | 997,808 | 190,076 | 19.05% | 448 | 44 | 9.82% | 28,640 | 22,411 | 78.25% |
| LEDA | Europe | 288,652 | 67,668 | 23.44% | 26 | 10 | 38.46% | 3,000 | > 2,956 | > 98.53% |
| FunAndes | Andes | 105,466 | 0 | 0% | 24 | 0 | 0% | 2,694 | 0 | 0% |
| MelastomaTRAITs | Neotropics | 44,738 | 7,294 | 16.30% | 66 | 45 | 68.18% | 2,520 | > 921 | > 36.55% |
| Rasgos-CL | Chile | 25,174 | 12,166 | 48.33% | 23 | 3 | 13.04% | 662 | > 607 | > 91.69% |
| China Plant Trait Database | China | 10,184 | 0 | 0% | 76 | 0 | 0% | 1,529 | 0 | 0% |
| EstablishMed | Mediterranean | 4,728 | 1,825 | 39% | 10 | 2 | 20% | 134 | / | / |
| **Database on plant regeneration traits** | | | | | | | | | | |
| SeedArc | Global | 47,000 | 47,000 | 100% | 1 | 1 | 100% | 4,500 | 4,500 | 100% |
| Lososová et al. 2023 | Global | 13,333 | 13,333 | 100% | 2 | 2 | 100% | 10,327 | 10,327 | 100% |
| SylvanSeeds | Global | 4,012 | 4,012 | 100% | 1 | 1 | 100% | 334 | 334 | 100% |
| Rosbakh et al. 2020 | Global | 2,690 | 2,690 | 100% | 3 | 3 | 100% | 2,890 | 2,890 | 100% |
| DiasMorph | Central Europe | 94,214 | 94,214 | 100% | 8 | 8 | 100% | 1,442 | 1,442 | 100% |
| Vaessen et al. 2023 | Guianas | 19,039 | 19,039 | 100% | 19 | 19 | 100% | 1,622 | 1,622 | 100% |
| Rock n’ Seeds | Brazil | 10,187 | 10,187 | 100% | 16 | 16 | 100% | 383 | 383 | 100% |
| Kimura et al. 2020 | Japan | > 516 | > 516 | 100% | 4 | 4 | 100% | 129 | 129 | 100% |

Total traits = all traits recorded in the database; seed traits = diaspore-related traits only; percentage = diaspore-related traits as a proportion of total traits; / = inaccessible data.

**References (ordered by the “Database” column)**

**Kattge J, Bönisch G, Díaz S, Lavorel S, Prentice IC, Leadley P, Tautenhahn S, Werner GDA, Aakala T, Abedi M *et al*. 2020.** TRY plant trait database – enhanced coverage and open access. *Global Change Biology* **26**: 119–188.

**Weigelt P, König C, Kreft H. 2020.** GIFT – A Global Inventory of Floras and Traits for macroecology and biogeography. *Journal of Biogeography* **47**: 16–43.

**Maitner BS, Boyle B, Casler N, Condit R, Donoghue IIJ, Durán SM, Guaderrama D, Hinchliff CE, Jørgensen PM, Kraft NJB *et al*. 2018.** The bien r package: A tool to access the Botanical Information and Ecology Network (BIEN) database. *Methods in Ecology and Evolution* **9**: 373–379.

**Komatsu KJ, Avolio ML, Padullés Cubino J, Schrodt F, Auge H, Cavender-Bares J, Clark AT, Flores-Moreno H, Grman E, Harpole WS *et al.* 2024.** CoRRE Trait Data: A dataset of 17 categorical and continuous traits for 4079 grassland species worldwide. *Scientific Data* **11**: 795.

**Falster D, Gallagher R, Wenk EH, Wright IJ, Indiarto D, Andrew SC, Baxter C, Lawson J, Allen S, Fuchs A *et al*. 2021.** AusTraits, a curated plant trait database for the Australian flora. *Scientific Data* **8**: 254.

**Kleyer M, Bekker RM, Knevel IC, Bakker JP, Thompson K, Sonnenschein M, Poschlod P, Van Groenendael JM, Klimeš L, Klimešová J *et al.* 2008.** The LEDA Traitbase: a database of life-history traits of the Northwest European flora. *Journal of Ecology* **96**: 1266–1274.

**Báez S, Cayuela L, Macía MJ, Álvarez-Dávila E, Apaza-Quevedo A, Arnelas I, Baca-Cortes N, Bañares de Dios G, Bauters M, Ben Saadi C, Blundo C *et al*. 2022.** FunAndes – A functional trait database of Andean plants. *Scientific Data* **9**: 511.

**Reginato M, Ordónez-Parra CA, Messeder JVS, Brito VLG, Dellinger A, Kriebel R, Marra C, Melo L, Cornelissen T, Fuzessy L, *et al.* 2024.** MelastomaTRAITs 1.0: A database of functional traits in Melastomataceae, a large pantropical angiosperm family. *Ecology* **105**: e4308.

**Alfaro E, Pérez-Tello V, Acevedo M, Ovalle J, Segovia R, Craven D. 2023.** Rasgos-CL: A functional trait database of Chilean woody plants. *Global Ecology and Biogeography* **32**: 2072–2084.

**Wang H, Harrison SP, Li M, Prentice IC, Qiao S, Wang R, Xu H, Mengoli G, Peng Y, Yang Y. 2022.** The China plant trait database version 2. *Scientific Data* **9**: 769.

**Acevedo-Limón L, Rumeu B, Bracho-Estévanez CA, González-Varo JP. 2024.** EstablishMed, a dataset of transition probabilities for woody plant establishment in the Mediterranean Region. *Global Ecology and Biogeography* **33**: e13879.

**Fern****ández-Pascual E, Carta A, Rosbakh S, Guja L, Phartyal SS, Silveira FAO, Chen S-C, Larson JE, Jiménez-Alfaro B. 2023.** SeedArc, a global archive of primary seed germination data. *New Phytologist* **240**: 466–470.

**Lososová Z, Axmanová I, Chytrý M, Midolo G, Abdulhak S, Karger DN, Renaud J, Van EJ, Vittoz P, Thuiller W. 2023.** Seed dispersal distance classes and dispersal modes for the European flora. *Global Ecology and Biogeography* **32**: 1485–1494.

**Fernández-Pascual E. 2021.** SylvanSeeds, a seed germination database for temperate deciduous forests. *Journal of Vegetation Science* **32**: e12960.

**Rosbakh S, Baskin CC, Baskin JM. 2020.** Nikolaeva et al.’s reference book on seed dormancy and germination. *Ecology* **101**: e03049.

**Dayrell RLC, Begemann L, Ott T, Poschlod P. 2024.** DiasMorph: a dataset of morphological traits and images of Central European diaspores. *Scientific Data* **11**: 781.

**Vaessen RW, van Wijngaarden K, Boeschoten L, Knippers R, Durazzo L, Verkuil L, van Kuijk M. 2023.** Fruit and seed traits and vertebrate–fruit interactions of tree species occurring in Guyana, Suriname, and French Guiana. *Ecology* **104**: e4165.

**Ordóñez-Parra CA, Dayrell RLC, Negreiros D, Andrade ACS, Andrade LG, Antonini Y, Barreto LC, Barros FV, Carvalho VC, Corredor BAD *et al*. 2023.** Rock n’ Seeds: A database of seed functional traits and germination experiments from Brazilian rock outcrop vegetation. *Ecology* **104**: e3852.

**Kimura MK, Mizuki I, Kawamura R, Koike M, Furumoto R, Kusumoto B, Fuji A, Kubota Y, Enoki T. 2020.** Seed size and weight of 129 tree species in Japan. *Ecological Research* **35**: 787–791.

**Table S2** Seed traits in the Chinese Seed Trait Database, ordered by descending records.

| **Trait name** | **Trait type** | **Number of records** | **Number of species** | **Format** |
| --- | --- | --- | --- | --- |
| Seed length | Morphological | 10,676 | 2,612 | Continuous |
| Seed mass | Morphological | 9,644 | 2,614 | Continuous |
| Seed width | Morphological | 9,434 | 2,270 | Continuous |
| Fruiting month | Phenological | 7,591 | 2,474 | Categorical |
| Fruit length | Morphological | 7,571 | 2,131 | Continuous |
| Fruit type | Morphological | 5,131 | 2,812 | Categorical |
| Fruit width | Morphological | 5,012 | 1,500 | Continuous |
| Plant height | Dispersal | 4,999 | 1,873 | Continuous |
| Seed thickness | Morphological | 4,315 | 1,100 | Continuous |
| Seed germination percentage | Physiological | 3,738 | 1,504 | Continuous |
| Seed colour | Morphological | 3,737 | 2,111 | Categorical |
| Fruit colour | Morphological | 2,685 | 1,223 | Categorical |
| Seed number per fruit | Quantitative | 2,327 | 1,049 | Continuous |
| Embryo length | Morphological | 2,259 | 714 | Continuous |
| Embryo width | Morphological | 2,021 | 544 | Continuous |
| Seed dehydration tolerance | Physiological | 1,609 | 841 | Categorical |
| Fruit mass | Morphological | 1,534 | 467 | Continuous |
| Seed aspect ratio | Morphological | 1,454 | 176 | Continuous |
| Cotyledon length | Morphological | 1,346 | 416 | Continuous |
| Cotyledon number per seed | Quantitative | 1,315 | 803 | Continuous |
| Cotyledon width | Morphological | 1,315 | 404 | Continuous |
| Seed dispersal mode | Dispersal | 1,245 | 637 | Categorical |
| Seed coat thickness | Morphological | 1,167 | 242 | Continuous |
| Fruit thickness | Morphological | 1,164 | 354 | Continuous |
| Seed dispersal month | Phenological | 1,147 | 601 | Categorical |
| Cotyledon thickness | Morphological | 1,115 | 287 | Continuous |
| Embryo thickness | Morphological | 1,100 | 265 | Continuous |
| Seed dormancy | Physiological | 1,060 | 814 | Categorical |
| Seed longevity | Physiological | 820 | 586 | Continuous |
| Endosperm | Morphological | 775 | 757 | Categorical |
| Cone length | Morphological | 740 | 131 | Continuous |
| Cone width | Morphological | 682 | 126 | Continuous |
| Seed moisture content | Chemical | 553 | 199 | Continuous |
| Seed germinability | Physiological | 535 | 93 | Continuous |
| Pericarp thickness | Morphological | 530 | 130 | Continuous |
| Fruit aspect ratio | Morphological | 483 | 79 | Continuous |
| Embryo colour | Morphological | 412 | 408 | Categorical |
| Seed wing length | Morphological | 311 | 58 | Continuous |
| Kernel mass | Morphological | 280 | 21 | Continuous |
| Cone mass | Morphological | 274 | 28 | Continuous |
| Endosperm colour | Morphological | 272 | 271 | Categorical |
| Seed wing width | Morphological | 271 | 50 | Continuous |
| Seed lipid content | Chemical | 253 | 47 | Continuous |
| Radicle length | Morphological | 247 | 175 | Continuous |
| Radicle width | Morphological | 235 | 168 | Continuous |
| Fruit fresh mass | Morphological | 222 | 82 | Continuous |
| Seed volume | Morphological | 222 | 22 | Continuous |
| Carpopodium length | Morphological | 215 | 26 | Continuous |
| Hypocotyl width | Morphological | 201 | 150 | Continuous |
| Hypocotyl length | Morphological | 192 | 144 | Continuous |
| Cone aspect ratio | Morphological | 181 | 18 | Continuous |
| Seed hair | Morphological | 179 | 177 | Categorical |
| Seed set percentage | Quantitative | 162 | 44 | Continuous |
| Fruit hair | Morphological | 159 | 98 | Categorical |
| Seed with appendage length | Morphological | 148 | 72 | Continuous |
| Seed production | Quantitative | 144 | 37 | Continuous |
| Seed yield percentage | Quantitative | 142 | 21 | Continuous |
| Seed protein content | Chemical | 140 | 18 | Continuous |
| Seed viability | Physiological | 138 | 41 | Continuous |
| Seed fresh mass | Morphological | 124 | 52 | Continuous |
| Seed germination time | Physiological | 124 | 21 | Continuous |
| Seed number per cone | Quantitative | 123 | 36 | Continuous |
| Hypocotyl thickness | Morphological | 117 | 92 | Continuous |
| Seed starch content | Chemical | 114 | 12 | Continuous |
| Seed coat mass | Morphological | 109 | 4 | Continuous |
| Kernel length | Morphological | 107 | 13 | Continuous |
| Kernel width | Morphological | 106 | 12 | Continuous |
| Pericarp mass | Morphological | 105 | 16 | Continuous |
| Kernel yield | Quantitative | 103 | 11 | Continuous |
| Kernel lipid content | Chemical | 95 | 15 | Continuous |
| Seed soluble sugar content | Chemical | 94 | 11 | Continuous |
| Radicle thickness | Morphological | 78 | 66 | Continuous |
| Seed surface area | Morphological | 76 | 11 | Continuous |
| Cone colour | Morphological | 75 | 18 | Categorical |
| Aril thickness | Morphological | 74 | 1 | Continuous |
| Seed with appendage width | Morphological | 66 | 35 | Continuous |
| Awn length | Morphological | 64 | 9 | Continuous |
| Fruit wing width | Morphological | 63 | 13 | Continuous |
| Fruit wing length | Morphological | 62 | 11 | Continuous |
| Seed wing thickness | Morphological | 61 | 6 | Continuous |
| Cone fresh mass | Morphological | 60 | 7 | Continuous |
| Kernel thickness | Morphological | 55 | 7 | Continuous |
| Fruit volume | Morphological | 36 | 6 | Continuous |
| Carpopodium thickness | Morphological | 33 | 6 | Continuous |
| Endosperm thickness | Morphological | 29 | 23 | Continuous |
| Fruit with appendage length | Morphological | 29 | 15 | Continuous |
| Seed dispersal distance | Dispersal | 27 | 9 | Continuous |
| Kernel moisture content | Chemical | 27 | 5 | Continuous |
| Seed phosphorus content | Chemical | 25 | 9 | Continuous |
| Cone thickness | Morphological | 24 | 3 | Continuous |
| Fruit moisture content | Chemical | 23 | 7 | Continuous |
| Seed nitrogen content | Chemical | 23 | 9 | Continuous |
| Seed carbon content | Chemical | 22 | 8 | Continuous |
| Fruit production | Quantitative | 21 | 4 | Continuous |
| Fruit surface area | Morphological | 16 | 2 | Continuous |
| Kernel colour | Morphological | 16 | 1 | Categorical |
| Seed number per flower | Quantitative | 16 | 1 | Continuous |
| Diaspore descent velocity | Dispersal | 15 | 14 | Continuous |
| Fruit number per plant | Quantitative | 15 | 7 | Continuous |
| Seed reducing sugar content | Chemical | 15 | 2 | Continuous |
| Fruit lipid content | Chemical | 13 | 2 | Continuous |
| Seed wing mass | Morphological | 13 | 2 | Continuous |
| Endosperm length | Morphological | 12 | 6 | Continuous |
| Cotyledon colour | Morphological | 11 | 11 | Categorical |
| Seed crude fibre content | Chemical | 11 | 2 | Continuous |
| Hypocotyl mass | Morphological | 11 | 1 | Continuous |
| Fruit wing thickness | Morphological | 10 | 3 | Continuous |
| Cone moisture content | Chemical | 10 | 1 | Continuous |
| Seed hair length | Morphological | 9 | 3 | Continuous |
| Fruit set percentage | Quantitative | 8 | 3 | Continuous |
| Cone volume | Morphological | 8 | 2 | Continuous |
| Pericarp fresh mass | Morphological | 8 | 2 | Continuous |
| Endosperm width | Morphological | 7 | 4 | Continuous |
| Carpopodium mass | Morphological | 7 | 1 | Continuous |
| Aril mass | Morphological | 7 | 1 | Continuous |
| Pericarp moisture content | Chemical | 5 | 1 | Continuous |
| Seed coat lipid content | Chemical | 5 | 1 | Continuous |
| Fruit protein content | Chemical | 3 | 1 | Continuous |

**Notes S1** Chinese summary of our database

植物性状是表征环境适应策略与资源分配模式的关键指标，为理解物种分布、群落构建及生态系统功能提供了重要基础。近年来，全球及区域尺度的植物性状数据库不断完善，推动了宏观生态学、功能生态学及生物多样性保护等领域的发展。然而，现有数据库主要聚焦于叶片等生长器官，种子、果实等繁殖器官的记录相对不足，形成显著的数据短板。此外，数据的地域分布极不均衡，部分生物多样性热点地区数据匮乏，进一步加剧了大尺度生态学研究中的地理偏倚问题。中国作为全球生物多样性热点地区之一，拥有逾3.5万种维管植物和多样化的生态系统，丰富的生物资源促使了大量本土研究的开展，相关成果刊载于众多中文期刊，积累了数量可观的植物传播体（diaspore）性状数据，却因语言障碍和获取渠道限制尚未被国际学界广泛利用。

为突破植物传播体性状数据在数量和地域分布上的局限，我们构建了中国种子性状数据库（Chinese Seed Trait Database, CSTD），系统整合了694个中文数据来源，包括681篇期刊论文、10本专著和3个在线数据库，共收录110451条数据记录，涵盖3897种植物（隶属1416属、214科），涉及118项性状，覆盖中国全部生物区系。数据库由三大核心模块构成：（1）采样地点信息，包括地名、地理坐标和气候数据；（2）物种信息，包括分类单元、生活型和生长型；（3）性状信息，包括性状名称、具体数据及其单位。其中，79%的记录包含地名信息，66.3%具备精确地理坐标，数据分布横跨45.6°纬度和6000 m海拔范围，具备显著的大尺度应用潜力。数据库广泛覆盖植物类群，其中接近40%的物种拥有10种以上性状记录，为系统发育比较分析提供基础。CSTD收录的性状涵盖形态、生理、化学、物候、数量及传播六大类，为全面描绘种子性状综合征和解析植物繁殖更新策略多样性提供了重要的数据支持。

总之，CSTD通过整合中文文献中海量而分散的传播体性状数据，有效填补了全球植物功能性状研究中的Raunkiæran短缺，为植物学、生态学和进化生物学的宏观研究提供了宝贵资源。未来，CSTD将持续更新和拓展，诚邀全球植物学家、生态学家及相关研究人员共同参与数据库的建设与完善，推动数据共享与集成，促进全球种子性状信息的全面整合与深入应用。
